# High Prevalence of Sarcopenia in Patients with Newly Diagnosed Gastroenteropancreatic Neuroendocrine Tumours (GEP-NETs), but No Association with the Risk of Surgical Complications

**DOI:** 10.3390/nu16223790

**Published:** 2024-11-05

**Authors:** Dominique S. V. M. Clement, Monique E. van Leerdam, Margot E. T. Tesselaar, Parthi Srinivasan, Krishna Menon, Koert Kuhlmann, Anne den Hartog, George Giovos, Martin O. Weickert, Rajaventhan Srirajaskanthan

**Affiliations:** 1 Kings Health Partners, ENETS Centre of Excellence, Institute of Liver Studies, King’s College Hospital, London SE5 9RS, UK; 2 Department of Gastroenterology, King’s College Hospital, London SE5 9RS, UK; 3 Department of Gastrointestinal Oncology, Netherlands Cancer Institute, ENETS Centre of Excellence, 1066 CX Amsterdam, The Netherlands; 4 Department of Gastroenterology and Hepatology, Leiden University Medical Center, 2333 ZA Leiden, The Netherlands; 5 Department of Hepatopancreaticobiliairy Surgery, King’s College Hospital, London SE5 9RS, UK; 6 Department of Gastrointestinal Surgery, Netherlands Cancer Institute, ENETS Centre of Excellence, 1066 CX Amsterdam, The Netherlands; 7 The ARDEN NET Centre, ENETS Centre of Excellence, University Hospitals Coventry and Warwickshire NHS Trust, Coventry CV2 2DX, UK; george.yovos@nhs.net (G.G.);

**Keywords:** gastropancreatic neuroendocrine tumour, sarcopenia, surgery

## Abstract

**Background:** Sarcopenia is a muscle disease that occur across a lifetime. It is commonly described in the aging population but can occur earlier in life in patients with cancer. Previous studies demonstrated sarcopenia is highly prevalent in patients with gastroenteropancreatic neuroendocrine tumours (GEP-NETs). In solid organ cancers, such as colorectal or pancreatic cancer, the presence of sarcopenia is associated with surgical complications. It is unknown if sarcopenia in patients with GEP-NETs is a risk factor for surgical complications. **Methods:** A multicentre retrospective study was performed in patients with a recently diagnosed GEP-NET and surgery to the primary tumour. CT scans were analysed for body composition analyses to assess for the presence of sarcopenia. Data regarding surgical procedures and complications were collected. Any major surgical complication was considered as Clavien–Dindo score ≥ 3. **Results:** This study included 180 patients, with 83 being male (46%) with a median age of 62 years (IQR 54–69). Most patients (n = 138, 77%) had a small intestinal NET, while 36 patients (20%) had pancreatic NETs. Sarcopenia was present in 109 patients (61%). In 43 patients (24%), surgical complications were recorded, and 21 complications (49%) were considered as major. Any type of surgical complication was not statistically different between patients without sarcopenia (n = 17, 24%) and with sarcopenia (n = 26, 24%)—a *p*-value of 0.36. This was the same for major complications; between patients without sarcopenia (n = 5, 24%) and with sarcopenia (n = 16, 76%)—a *p*-value of 0.18. **Conclusions:** Sarcopenia is highly prevalent in patients with a recently diagnosed GEP-NET, but this is not associated with major surgical complications. Future studies should include pathophysiological mechanisms that could be used to identify the causes of sarcopenia, its effect on quality of life and other oncological outcomes.

## 1. Introduction

Neuroendocrine neoplasms are a group of uncommon cancers that can be subdivided into well-differentiated neuroendocrine tumours (NETs) and poorly differentiated neuroendocrine carcinomas (NECs). NET and NEC cancers should be seen as different cancers due to their different treatment options and prognoses. NETs can be classified based on the mitotic count and/or the Ki-67 index into grade 1 (G1, mitotic count less than 2/2 mm^2^ and Ki-67 less than 3%), grade 2 (G2, mitotic rate 2–20/2 mm^2^ and Ki-67:3–20%) or grade 3 (G3, mitotic rate greater than 20/2 mm^2^ and Ki-67 index greater than 20%) [1]. NETs originate from enterochromaffin cells and are predominantly located within the pulmonary or gastroenteropancreatic (GEP) tract, with the small intestine and pancreas being the most prevalent sites [2]. GEP-NETs may secrete hormones that enter systemic circulation, leading to clinical symptoms; such tumours are classified as functional NETs [3].

Due to their localization within the GEP tract, NETs can cause nonspecific symptoms such as abdominal pain, diarrhoea and weight loss [4,5]. These nonspecific symptoms can lead to a delay in diagnosing a GEP-NET, with some studies reporting up to 50% of patients having symptoms for 2–5 years prior to being diagnosed [6,7]. Due to these symptoms and the prolonged period before a diagnosis is made, patients are at risk of malnutrition.

There are many different types of nutrition disorders, including malnutrition, underweight, sarcopenia and cachexia [8]. Sarcopenia refers to adverse changes in muscle mass and muscle function that occur over a lifetime. While it is frequently observed in older adults, it can also manifest earlier in life, particularly in patients with cancer [9]. A recent publication from our group revealed that 75% of patients with GEP-NETs receiving treatment with somatostatin analogues (SSAs) meet the Global Leadership Initiative on Malnutrition (GLIM) criteria for malnutrition [10]. Furthermore, approximately 70% of these patients had sarcopenia [11].

There are multiple techniques that can be used for diagnosing sarcopenia, but the golden standard is considered body composition analysis using computer tomography (CT) or magnetic resonance imaging (MRI) scan images [9]. Body composition analysis can also be used to analyse a person’s fat mass and to diagnose adipopenia, which pertains fat mass depletion [12]. It can also be utilised for the diagnosis of myosteatosis, which encompasses fat infiltration into the muscle and is considered a marker for muscle quality [13].

Studies examining gastrointestinal cancers, such as colorectal and pancreatic cancer, have demonstrated that the presence of sarcopenia is associated with an increased risk of postoperative surgical complications [14,15,16,17,18,19]. Two recent studies have reported sarcopenia to be present in 67–87% of patients with a NEN [20,21]. However, none of the studies investigated if there is an association between sarcopenia and postoperative complications in patients with GEP-NETs.

The aim of this study is to assess the presence of sarcopenia in a cohort of patients with recently diagnosed GEP-NETs with surgical resection for their primary tumour. Secondly, we explore if sarcopenia is associated with surgical complications.

## 2. Materials and Methods

### 2.1. Study Design and Study Population

A multi-centre retrospective study was performed in three hospitals, Netherlands Cancer Institute (NKI), Amsterdam, The Netherlands; the Arden NET Centre (ANC), Coventry, United Kingdom; and King’s College Hospital (KCH), London, the United Kingdom, which are all European Neuroendocrine Tumour Society (ENETS) centres of excellence. Eligible patients were identified from local prospectively maintained NET databases from each hospital.

Inclusion criteria: Adults (≥18 year) diagnosed with any grade or stage GEP-NETs in the period from 2006 to 2018 with a (low-dose) CT scan of the abdomen within 3 months of surgical resection of the primary tumour. The initial CT scan was performed either at a local hospital or at one of the participating centres where a NET was suspected. The NET diagnosis was confirmed via a histopathological examination. Weight, height and body mass index (BMI) were available.

Exclusion criteria: no availability of a CT scan of the abdomen at diagnosis, previous transplantation of liver or kidney, another form of any other solid-organ cancer within 1 year of diagnosis of GEP-NET, and ascites or spinal metal implants present.

There is an overlap of 102/114 patients with stage IV disease who were also included in a previous publication regarding the association of sarcopenia at diagnosis of a stage IV GEP-NET with overall survival [22]. However, the current study has a different research question and aim; therefore, the authors felt that the patients were suitable to be included.

### 2.2. Objectives

The primary objective was to describe the percentage of sarcopenia in patients with GEP-NETs and surgical resection of the primary NET. The secondary objective was to explore sarcopenia as a risk factor for postoperative complications.

### 2.3. Data Collection

**Figure 1:**
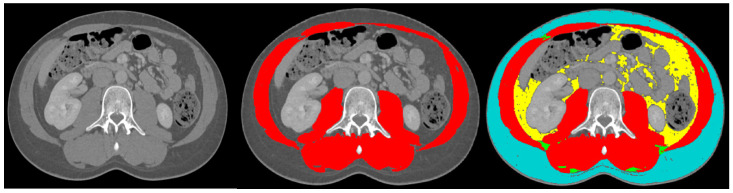
Example body composition analysis. The left image displays a cross-sectional view at the lumbar level L3, the middle image indicates muscle mass tagged in red, and the right image shows subcutaneous adipose tissue tagged in blue and visceral adipose tissue tagged in yellow.

The following data were collected: baseline demographic characteristics, height, weight and BMI. The WHO 2019 classification was used for the histological confirmation of grading and staging of the GEP-NET [1]. There are 4 categories of weight based on BMI: <18.50 kg/m^2^ is underweight, 18.51–24.99 kg/m^2^ is normal weight, 25.00–29.99 kg/m^2^ is overweight and >30 kg/m^2^ is obese [23]. Primary tumours were categorised as located in the small intestine (including duodenum), pancreas or other. For staging, the Tumor Node Metastasis (TNM) system was used, originating from the Union for International Cancer Control (UICC), 8th edition (2016). The American Society of Anaesthesiologists Classification (ASA) was used for vitality [24]. Surgical procedures were categorised to the small intestine (including all surgery to the colon), pancreas and other. The Clavien–Dindo classification for surgical complications was used [25], and a score of ≥3 was considered as a major surgical complication and considered as clinically relevant. Surgical complications were categorised into infectious (chest infection/pneumonia, skin infection, urinary tract infection, abscess), ileus (including gastropareses), anastomosis-related (leakage, stenosis) and other. Surgical complications within 30 days following surgery were included. For body composition analysis, a CT scan suggesting the presence of a NET was analysed using a single slice at lumbar level L3. Previous research has suggested that this area corresponds well with entire body muscle and fat mass [26,27]. The body composition analyses were performed using Slice-O-Matic software (5.0 Rev-8, Tomovision, Milletta, Magog, QC, Canada). The muscle and fat areas were identified and marked with a colour-coded label. These colour-coded labels were based on Hounsfield units (HU) for muscle, −29 HU to +150 HU; for subcutaneous adipose tissue, −150 HU to −50 HU; and for visceral adipose tissue, −190 HU to −30 HU. Figure 1 provides an example of body composition analysis. Sarcopenia was present if the skeletal muscle index was <53 cm^2^/m^2^ if BMI > 25 kg^2^/m^2^ or <43 cm^2^/m^2^ if BMI < 25 kg^2^/m^2^ for males and <41 cm^2^/m^2^ for females [9,23]. Myosteatosis was present if the muscle attenuation was <33 HU if BMI > 25 or <41 if BMI < 25 for both males and females [23]. The subcutaneous and visceral adipose tissue areas were added together to obtain the total adipose tissue area (TAT); adipopenia was present if males had TAT < 364 cm^2^ and females had TAT < 318 cm^2^ [28].

### 2.4. Statistical Analysis

For statistical analysis, SPSS version 29 (IBM, New York, NY, USA) was used. Continuous data were displayed as a median with an interquartile range and categorical data as a number with a percentage. The Mann–Whitney U test and chi-squared test were performed to describe differences between groups. To explore the association between any surgical complications and major surgical complications and sex, age, ASA classification, primary location, tumour stage and presence of sarcopenia, univariate logistic regression analyses were performed. Factors significant in the univariate analysis were included in the multivariate logistic regression analysis. A *p*-value of <0.05 was considered significant.

## 3. Results

### 3.1. Primary Outcome

This research identified 180 patients with a resected primary tumour and a CT scan available within 3 months prior to surgical intervention. A total of 83 male patients (46%) with a median age of 62 years (IQR 54–69) were included. Most patients (n = 138, 77%) had a small intestinal NET, and 116 patients (64%) had stage IV disease. Table 1 provides an overview of baseline characteristics. Within the subgroup of patients with a pancreatic primary NET, a total of 23 patients (64%) had a Whipple procedure or pylorus-preserving pancreaticoduodenectomy (PPPD), and 13 patients (36%) had a distal pancreatectomy.

Body composition analysis revealed the presence of sarcopenia in 109 patients (61%), adipopenia in 101 patients (56%) and myosteatosis in 77 patients (43%). Sarcopenia is most often present in patients who are underweight n = 6 (75%) or with normal weight n = 50 (69%) (Table 2).

Patients with sarcopenia are significantly older (*p*-value, 0.04), median age of 63 years (IQR 55–73), compared to patients without sarcopenia, median age of 61 years (IQR 52–67). Patients with sarcopenia have a significantly (*p*-value, 0.005) more advanced disease (stage IV n = 79, 68%) versus patients without sarcopenia (stage IV n = 37, 32%). In patients with a small intestinal NET, sarcopenia was present in 90 patients (65%), compared to patients with a pancreas NET, in whom 17 had sarcopenia (47%)—a *p*-value of 0.06. Patients with carcinoid syndrome show a trend towards more frequently sarcopenia with a *p*-value 0.06. 

### 3.2. Secondary Outcome—Surgical Complications

There were postoperative complications recorded in 43 patients (24%); details are displayed in Table 2. There were no statistically significant differences in sex, age, staging or grading between patients without surgical complications and those with complications. In patients with a pancreatic NET, n = 14 (39%) developed postoperative complications, of which n = 5 (36%) had a major complication. Regarding patients who had small bowel surgery, n = 28 (30%) had a surgical complication, of which n = 15 (54%) had a severe one. The presence of sarcopenia (*p*-value, 0.73), myosteatosis (*p*-value, 0.35) or adipopenia (*p*-value, 0.53) was not significantly different between patients without or with surgical complications.

In univariate analysis, no risk factors (including sarcopenia) could be identified to have a relationship with the presence of major surgical complications, as demonstrated in Table 3. Correcting for age and sex, sarcopenia is not a risk factor for major surgical complications according to multivariate analysis (Table 3).

## 4. Discussion

This study describes sarcopenia as being present in 61% of patients with a newly diagnosed GEP-NET and having surgical resection from their primary tumour. No association was found between sarcopenia and major surgical complications.

The prevalence of sarcopenia at 61% at the diagnosis of a GEP-NET has been described in other publications in patients with GEP-NETs. However, there are differences when comparing our findings to other studies, such as Chan et al., who only included 49 patients with a progressive NET and reported a presence of sarcopenia in 67% of them [21]. Moreover, the study from Herrera et al. included 104 patients with newly diagnosed GEP-NET, with the prevalence of sarcopenia being 87% [20]. A study from our group regarding patients with metastatic GEP-NETs showed that sarcopenia was present in 69% of them [22]. Studies regarding patients with a recently diagnosed colorectal carcinoma or pancreatic carcinoma showed that the presence of sarcopenia varies ranges from 12 to 60% and from 24 to 78%, respectively. This wide variance in the presence of sarcopenia could be explained by the different cut-offs that these studies used. For example, the study by van Rijssen et al. did not correct for the BMI, while our study and other studies did [14,16,29,30]. The high presence of sarcopenia in our study could be explained by the period of symptoms preceding the diagnosis of a NET. Gastrointestinal symptoms may lead to a reduced nutritional intake. A NET itself can result in a systemic inflammatory response and cytokine release, which influences the developing sarcopenia [4,5,6,7]. As 64% of the patients in this study had stage IV disease, the disease burden was high, which could have contributed to cytokine release, thus driving sarcopenia.

The median BMI of patients in this study was 25.5. However, in patients who were overweight or obese, sarcopenia was present in 62% and 43% of them, respectively. This has been described in other publications when assessing patients with solid organ cancer in their pancreases or colons [16,31,32,33,34]. The current criteria for sarcopenia include BMI as a diagnostic criterium for male patients only; when the male patient has a BMI above 25 kg/m^2^ sarcopenia is present when the skeletal muscle index is below 53 cm^2^/m^2^, while when the BMI is below 25 kg/m^2^ sarcopenia is present when the skeletal muscle index is below 43 cm^2^/m^2^. For female patients, there is no BMI-related diagnostic criterium for the skeletal muscle index. Therefore, there could be a risk of missing female patients with obesity and sarcopenia, as a higher BMI in male patients has a low skeletal muscle index cut-off. Furthermore, it is important to note that the prevalence of obesity is rising within the general population; therefore, assessing people’s nutritional status solely based on the body mass index (BMI) is insufficient [35] and will risk missing the diagnosis of sarcopenia, especially in women. The current data suggest that BMI alone is not enough to measure a patient’s nutritional status and should include weight loss and sarcopenia [10].

In this study, the overall complication rate following surgery was 24%, and the major complication rate was 12%. For patients who had pancreas surgery, the complication rate was 39% and the major complication rate 14%. Comparing the complications for the patients who underwent a pancreatic surgical intervention with patients in a systematic review and meta-analysis, the complication rate was reported to be 14–58%. However, no difference in the severity of complications was reported [36]. Interestingly, when comparing pancreas surgery for a NET, it has a lower reported complication rate compared to surgery for a pancreatic adenocarcinoma. In a study from the United Kingdom by Sandini et al., an overall complication rate of 66% was reported for patients undergoing surgery for a pancreatic adenocarcinoma, with the major complication rate being 34–47% [16]. However, in the study by Sandini et al., all patients underwent a pancreatoduodenectomy; on the other hand, in the systematic review and meta-analysis, there was a mix of patients who had an enucleation of a pancreatic tumour, distal pancreatectomy, central pancreatectomy or pancreatoduodenectomy [16,36]. The lower complication rate observed in the current study may be attributed to the limited number of patients undergoing pancreatic surgery, which constituted 20% of the study cohort, in contrast to small intestinal surgery, which accounted for 70% of the population. The complication rate for patients who underwent surgery to the small intestine was 30%, and the major complication rate was 11%. A recent published systematic review and meta-analysis regarding complications following surgery for small intestine NETs reported a complication rate of 15% and a major complication rate of 9%, which is in line with our findings [37]. This low risk of complications could be an explanation for the lack of association with sarcopenia in patients with small intestinal NETs. Another explanation for the relatively low severe complication rate of 12% could be the surgical centre, as all patients underwent surgery in ENETS Centers of Excellence.

In the current study, sarcopenia was not a risk factor for major surgical complications. The aforementioned systematic reviews and meta-analyses regarding pancreatic or small intestinal surgery for NETs did not include body composition analysis [36,37]. The only comparable studies regarding sarcopenia and patients with NETs did not account for surgical complications [20,21]. Our findings are discrepant in comparison to studies that investigated colorectal and pancreatic adenocarcinomas. There are studies that studied colorectal cancer and sarcopenia that reported more frequent surgical complications or major complications [14,29]. In contrast, some studies reported no increased risk [34]. Another study investigating colorectal cancer reported myosteatosis to be significantly associated with complications [17]. Two studies focusing on sarcopenia in patients with pancreatic cancer could not correlate this factor with (major) surgical complications. One of these studies found adipopenia to be a risk factor for surgical complications in a univariate analysis, but this could not be confirmed in multivariate analysis [16]. Intriguingly, the other study found myosteatosis to be a risk factor for surgical complications in uni- and multivariate analyses [30]. All the mentioned studies, including this study, are retrospective studies with a high prevalence of sarcopenia and variable rates of surgical complications. A prospective study including all patients that underwent surgery for a NET might help to elucidate the role of sarcopenia in relationship to surgical complications.

In the current study, 64% of patients had metastases from their NETs and had to undergo surgical removal of their primary tumour, which was located in the small bowel in most of the patients. Several studies on the removal of the primary tumour in patients with metastatic small intestinal located NETs have reported conflicting results. There are studies that demonstrate a survival benefit when removing the primary small intestinal tumour, but there are also studies that failed to demonstrate this in patients who did not display any symptoms [38,39,40]. A recent study that included patients with asymptomatic metastatic small bowel NETs showed that resection of the primary tumour is an independent risk factor for disease-specific mortality but not for overall survival [41]. Cases of metastases concerning resection of the primary pancreas tumour lack evidence [42].

Myosteatosis was prevalent in 43% of our patients, which is in line with findings of Chan et al., wherein it was present in 71% of their patients. The difference in the presence of myosteatosis could be explained due to the different patients included in the studies, as Chan et al.’s study, where all patients had progressive disease. It could be hypothesized that progressive disease results in an ongoing systemic inflammatory response, cytokine release, decrease in muscle mass and increase in intermuscular fat measured in patients with myosteatosis [21].

This study has several limitations, with the primary one being its retrospective design. It could be said that surgical complications have not been reported clearly in patient records or could be missed as some patients might have went to their local hospital with postoperative complications and not to the study centre. On the other hand, our primary outcome was the association between major complications, for which management nearly always encompasses surgery in the hospital. A correlation with weight loss and gastrointestinal symptoms is missing. Other parameters for the diagnosis of sarcopenia, such as handgrip strength and physical performance, are lacking.

Future research should focus on pathophysiological mechanisms that could be used to identify the causes of sarcopenia. Once established, the role of sarcopenia and its effects on quality of life and other oncological outcomes should be investigated [43,44].

## 5. Conclusions

Patients with a newly diagnosed GEP-NET scheduled for surgical resection of the primary tumour have a high prevalence of sarcopenia. However, sarcopenia is not a risk factor for the occurrence of major surgical complications.

## Figures and Tables

**Table 1:** Baseline characteristics and (*) differences between patients with and without sarcopenia. IQR, interquartile range; G1, grade 1; G2, grade 2; G3, grade 3.

	All Patients N = 180	No Sarcopenia N = 71	Sarcopenia N = 109	*p*-Value (*)
**Sex**	83 (46%)	37 (45%)	46 (55%)	0.19
**Age** (median, IQR)	62 (54–69)	61 (52–67)	63 (55–73)	**0.04**
**ASA classification**				0.32
I, n (%)	55 (31%)	18 (33%)	37 (67%)	
II, n (%)	98 (54%)	44 (45%)	54 (55%)	
III, n (%)	11 (6%)	5 (45%)	6 (55%)	
Missing, n (%)	16 (9%)	4 (25%)	12 (75%)	
**Primary location**				0.06
Small intestine, n (%)	138 (77%)	48 (35%)	90 (65%)	
Pancreas, n (%)	36 (20%)	19 (53%)	17 (47%)	
Other, n (%)	6 (3%)	4 (67%)	2 (33%)	
**Grading**				0.19
G1, n (%)	120 (67%)	53 (44%)	67 (56%)	
G2, n (%)	49 (27%)	16 (33%)	33 (67%)	
G3, n (%)	1 (1%)	1 (100%)	0	
Missing	10 (6%)	1 (10%)	9 (90%)	
**Carcinoid syndrome**, n (%)	63 (37%)	19 (30%)	44 (70%)	0.06
**Stage**				**0.005**
I, n (%)	3 (2%)	3 (100%)	0	
II, n (%)	24 (13%)	15 (62%)	9 (38%)	
III, n (%)	32 (18%)	14 (44%)	18 (56%)	
IV, n (%)	116 (64%)	37 (32%)	79 (68%)	
Missing, n (%)	5 (3%)	2 (40%)	3 (60%)	
**Days of hospital stay** (median, IQR)	9 (7–13)	9 (7–13)	9 (7–14)	0.79
**BMI categories**				**0.04**
Underweight, n (%)	8 (4%)	2 (25%)	6 (75%)	
Normal weight, n (%)	72 (40%)	22 (31%)	50 (69%)	
Overweight, n (%)	48 (27%)	18 (38%)	30 (62%)	
Obese, n (%)	51 (28%)	29 (57%)	22 (43%)	
Missing	1 (1%)	0	1 (100%)	
**Sarcopenia**, n (%)	109 (61%)			
**Myosteatosis**, n (%)	77 (43%)	23 (30%)	54 (70%)	**0.02**
**Adipopenia**, n (%)	101 (56%)	32 (32%)	69 (68%)	**0.009**

**Table 2:** Details of surgical complications.

	All Patients N = 180	No Sarcopenia N = 71	Sarcopenia N = 109	*p*-Value
**No complications**, n (%)	125 (69%)	48 (38%)	77 (62%)	
**Complications**				0.36
Infectious, n (%)	17 (9%)	7 (41%)	10 (59%)	
Ileus, n (%)	3 (2%)	1 (33%)	2 (67%)	
Anastomosis-related, n (%)	17 (9%)	5 (29%)	12 (71%)	
Other, n (%)	6 (3%)	4 (66%)	2 (34%)	
**Missing, n (%)**	14 (8%)			
**Minor complication**, n (%)	22 (12%)	12 (55%)	10 (45%)	0.18
**Major complication**, n (%)	21 (12%)	5 (24%)	16 (76%)	0.12

**Table 3:** Univariate and multivariate analysis risk factors for major surgical complications.

	Univariate Analysis		Multivariate Analysis	
	OR (95% CI)	*p*-Value	OR (95% CI)	*p*-Value
**Sex**	1.06 (0.42–2.66)	0.91	1.01 (0.4–2.59)	0.98
**Age**	0.98 (0.94–1.02)	0.24	0.97 (0.93–1.01)	0.16
**ASA classification**	0.5 (0.21–1.22)	0.13		
**Primary location**	0.94 (0.36–2.49)	0.90		
**Tumour stage**	1.59 (0.76–3.33)	0.22		
**Sarcopenia**	2.27 (0.79–6.53)	0.13	2.5 (0.86–7.32)	0.09

## Data Availability

All data generated during this study are included in this article. Further enquiries can be directed to the corresponding author.
